# Trans-scale live-imaging of an E5.5 mouse embryo using incubator-type biaxial light-sheet microscopy

**DOI:** 10.26508/lsa.202402839

**Published:** 2025-01-15

**Authors:** Go Shioi, Tomonobu M Watanabe, Junichi Kaneshiro, Yusuke Azuma, Shuichi Onami

**Affiliations:** 1 Laboratory for Comprehensive Bioimaging, RIKEN Center for Biosystems Dynamics Research (BDR), Kobe, Japan; 2 Laboratory for Developmental Dynamics, RIKEN Center for Biosystems Dynamics Research (BDR), Kobe, Japan; 3 Department of Stem Cell Biology, Research Institute for Radiation Biology and Medicine, Hiroshima University, Hiroshima, Japan

## Abstract

During mouse embryonic development, the embryonic day (E) 5.5 stage represents a crucial period for the formation of the primitive body axis, where the symmetry breaking of cellular states influences the multicellular system. Elucidating the detailed mechanisms of this process necessitates a trans-layered dynamic observation of the embryo and all internal cells. In this report, we present our success in achieving in-toto single-cell observation in a whole hemisphere of an E5.5 embryo for 12 h, using a newly developed incubator-type biaxial light-sheet microscope. To achieve the success, we optimized our microscope system, including an incubator for culture stability, and refining the observation protocol to reduce phototoxicity. Our key discovery is that the scan speed during light-sheet formation plays a critical role in reducing phototoxicity, rather than the irradiation intensity or the interval time between frames. This innovative system not only enabled in-toto single-cell tracking but also led to the discovery of the abrupt shrinking of embryos whose contractile center was located at the extraembryonic ectoderm during monotonous growth up to the E6.5 stage.

## Abstract (precis)

We achieve in-toto single-cell observation in a whole hemisphere of an E5.5 embryo for 12 h, optimizing a special microscope system, including an incubator, and refining the observation protocol.

## Introduction

The development of early mouse embryos, where all body parts, except for a section of the gut, originate from epiblast cell descendants (1), demands precise spatiotemporal control. Notably, on the embryonic day (E) 5.5, the cylindrical embryo assumes the most primitive body axis, the anteroposterior (A-P) axis, serving as the foundation for subsequent morphogenesis (2). A crucial aspect of A-P axis formation involves asymmetric cell migration, known as distal visceral endoderm (DVE) migration, occurring over ∼5 h (3). This process subsequently gives rise to the chirality of embryo morphology and/or signaling cascade (4, 5). Long-term single-cell tracking in the epiblast during DVE migration, coupled with the observation of tissue formation, is essential to unveil the mechanism by which the symmetry breaking of cellular states influences the multicellular system during embryonic development.

Advancements in optical microscopy specifications have contributed to successful long-term observations before the blastocyst stage (6, 7) or after E6.5 (8, 9) at single-cell resolution. However, until now, only one group has achieved single-cell observation in a living E5.5 embryo, with the duration of this observation limited to 90 min (8, 10).

We speculated that the practical challenge of limited observation duration stemmed from the instability of embryo culture on the microscope. Despite the optimization of culture conditions for the development of E5.5 embryos in a floor-standing incubator (11), maintaining temperature stability on a microscope proves challenging. Another factor restricting observation duration is the phototoxicity induced by continuous laser excitation on the embryo. Notably, the production of reactive oxygen species (ROS) is a key mechanism of phototoxicity during fluorescent observation (12). Although phototoxicity to stem cells is a consideration for many biological researchers, systematic investigations on this topic are limited. A recent study reported that longer wavelengths do not necessarily imply reduced toxicity, emphasizing the wavelength-dependent phototoxicity effect on in-vitro fertilization (13). This literature conveys a strong message that the phototoxicity effect on stem cells is not a simple phenomenon but rather multifaceted, necessitating careful considerations for the live imaging of a developing embryo.

In this study, we achieved uninterrupted simultaneous tracking of single-cell migration and the overall morphological changes in a living E5.5–E6.0 mouse embryo. This success resulted from the development of an incubator integrated into a microscope, specifically optimized for observing mouse embryo development, coupled with an investigation into phototoxicity under various observation conditions. The implemented system allowed the identificaion of all single cells in a hemisphere of the embryo, categorizing them into the epiblast, visceral endoderm, and DVE. Furthermore, we discovered a novel hiccup-like behavior, characterized by the abrupt shrinking of the embryo during monotonous growth. This article details the process from constructing the microscope system, investigating phototoxicity, to achieving in-toto single-cell tracking in a living E5.5 embryo.

## Results and Discussion

### Microscope system construction

The sample under investigation was an embryo from a mouse line expressing green fluorescent protein–tagged histone 2B ubiquitously (R26-H2B-EGFP mouse line) (14). Given that left–right asymmetry emerges after the E7.5 stage, we focused on observing one hemisphere of the embryo to study A-P axis formation. The required microscopic specifications were set as a goal as follows: a three-dimensional (3D) observation field of view measuring 0.3 × 0.3 × 0.15 mm³, a spatial resolution of less than 5 μm to individually resolve single nuclei, and a frame rate of less than 10 min to detect cell division over a 12-h period, considering an estimated embryo size of ∼300 μm.

**Figure 1.**
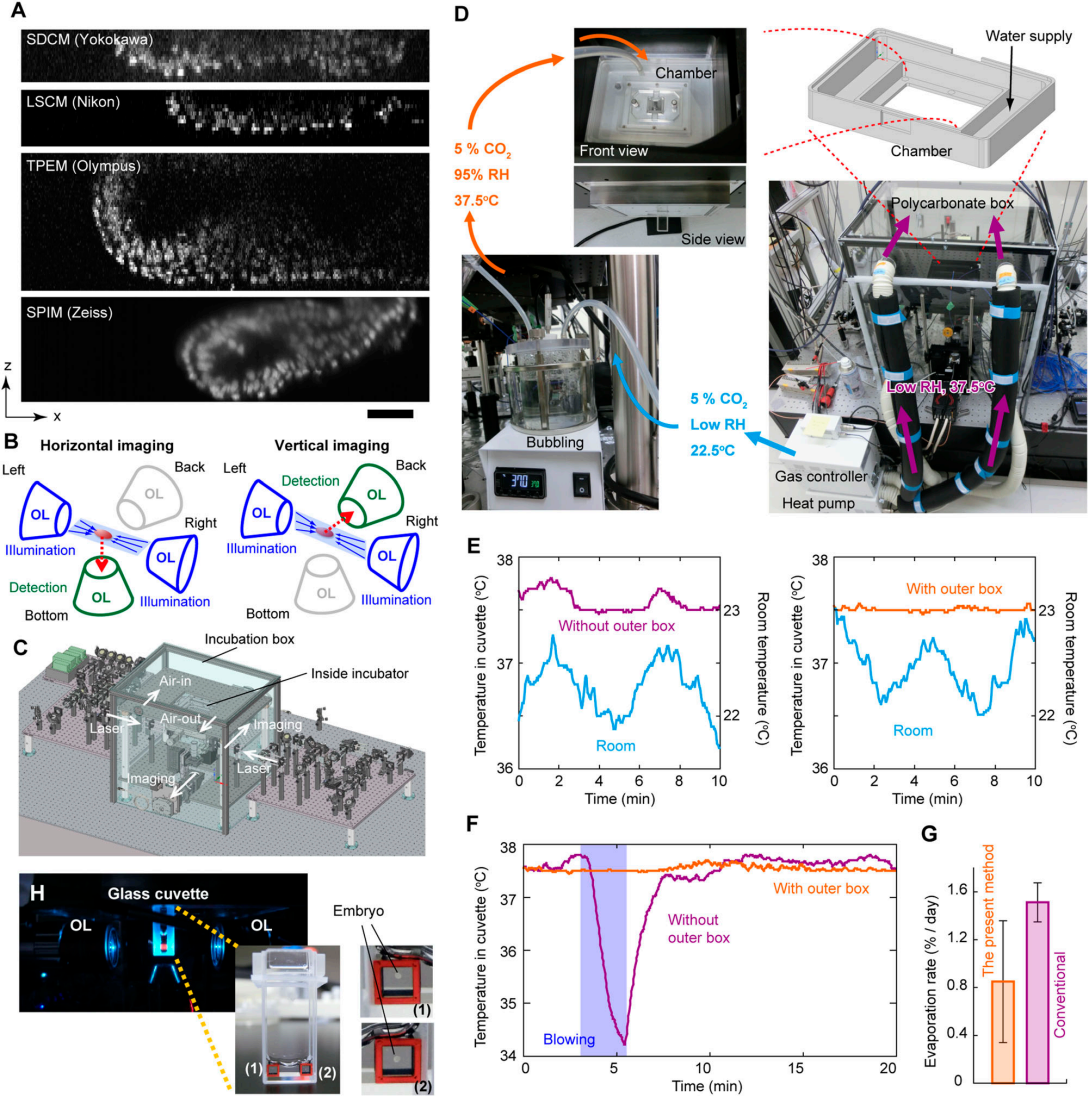
Incubator-type microscopic system for in-toto single-cell observation of a mouse E5.5 embryo. **(A)** Comparative analysis of E5.5 or E6.5 mouse embryo observations using four conventional microscopy techniques. Images of mouse embryos expressing H2B-GFP were acquired through SDCM (CV1000; Yokokawa), LSCM (A1; Nikon), TPEM (FV1000-MPE; Olympus), and mSPIM (Z1; Zeiss). The scale bar is 100 μm. **(B)** Schematic representation of objective placement for diSPIM. **(C)** Computer-aided design illustration depicting the entire system. **(D)** Schematic illustration and photographs illustrating air heating, bubbling, and loading system in the two-layered incubation system. **(E)** Time course depicting the temperature of the medium inside the cuvette without (*left*) and with (*right*) the polycarbonate box. Cyan represents room temperature; magenta or orange denotes medium temperature. **(F)** Time course of the temperature of the medium inside the cuvette without (*magenta*) and with (*orange*) the polycarbonate box and human-induced blowing (*blue*). **(G)** Measurement of medium evaporation rate (N = 10, Astec APM-30DR) in a commercial conventional incubator (*magenta*) and the two-layered incubation system (*orange*, N = 4). **(H)** Photograph of the glass cuvette, with embryos immobilized within each collagen-filled cube (*right panels*).

Before constructing the new microscope system, we conducted preliminary observations on the embryo using four different types of commercialized microscopes: spinning-disk confocal microscopy (SDCM), laser scanning confocal microscopy (LSCM), two-photon excitation microscopy (TPEM), and multidirectional selective plane illumination microscopy (mSPIM) (Fig 1A).

Observations revealed that single-photon excitation with LSCM or SDCM failed to visualize cells located deeper than 50 μm from the embryo surface. TPEM, on the contrary, demonstrated an observation depth of over 200 μm. Although mSPIM could also visualize cells at depths exceeding 200 μm, the image quality suffered as depth increased because of blurring and/or shadowing caused by the lens effect and/or random light scattering by cellular microstructures compared with TPEM.

Although TPEM’s temporal resolution was generally insufficient for tracking cell migration movements, the parallel use of SDCM could address this limitation. However, this approach necessitated significantly higher excitation energy; for instance, GFP observation was confined to an ∼40 μm^2^ field of view with a laser power of 1.56 W (15). Given these considerations, we chose mSPIM as the foundation for our new microscope system in this study.

The mSPIM, a specialized iteration of SPIM designed to mitigate shadowing artifacts (16), failed to eliminate image quality deterioration sufficiently for the isolation of all single cells at deeper sites (Fig 1A, SPIM). To overcome this problem, we integrated mSPIM with dual-view inverted SPIM (diSPIM) to achieve precise 3D rendering. This innovative approach allowed for the decomposition of single-cell resolution along one axis, even when not achieved in that specific axis (17). Two multidirectional laser sheets were introduced to the embryo from both sides and rotated by 90° for dual-axis acquisition (Fig 1B, blue, and Fig 1C). Two image detectors were strategically positioned to sequentially acquire transverse/horizontal and longitudinal/vertical images (Fig 1B, green).

To ensure a stable culture environment for embryonic development, our microscope system incorporated a two-layered incubator design. The microscope, excluding the optics for laser illumination, was encased in a polycarbonate box (Fig 1C and D). A small chamber was affixed to the microscope stage, housing the sample cuvette (Fig 1D and H). We conducted an investigation into the impact of this two-layered structure on temperature stability within the cuvette on the microscope stage.

Room temperature exhibited fluctuations within ± 0.5°C with a specific frequency, likely attributed to the room’s air conditioner (Fig 1E, left, cyan). The temperature of the medium in the cuvette mirrored this fluctuation, reaching up to 37.8°C (Fig 1E, left, magenta). The polycarbonate box effectively blocked heat exchange between the room and chamber, mitigating temperature fluctuations (Fig 1E, right).

Despite the small size of the chamber, the medium temperature in the cuvette proved sensitive to heat transfer from airflow caused by human operation (Fig 1F, magenta). The polycarbonate box substantially suppressed this sensitivity (Fig 1F, orange). Although the compact chamber size could potentially accelerate medium evaporation, the two-layered incubator was more effective in inhibiting evaporation compared with a commercialized incubator (Fig 1G). Furthermore, to maintain the normal developmental morphology of E5.5 embryos (14), embryos were embedded in a 3-mm cubic structure made of polycarbonate filled with collagen I gel (Fig 1H). This cube was securely affixed to the bottom of the cuvette using the surface tension of 150–200 μl of medium.

### Investigation of phototoxicity to embryonic stem cells (ESCs) during laser scanning

**Figure 2.**
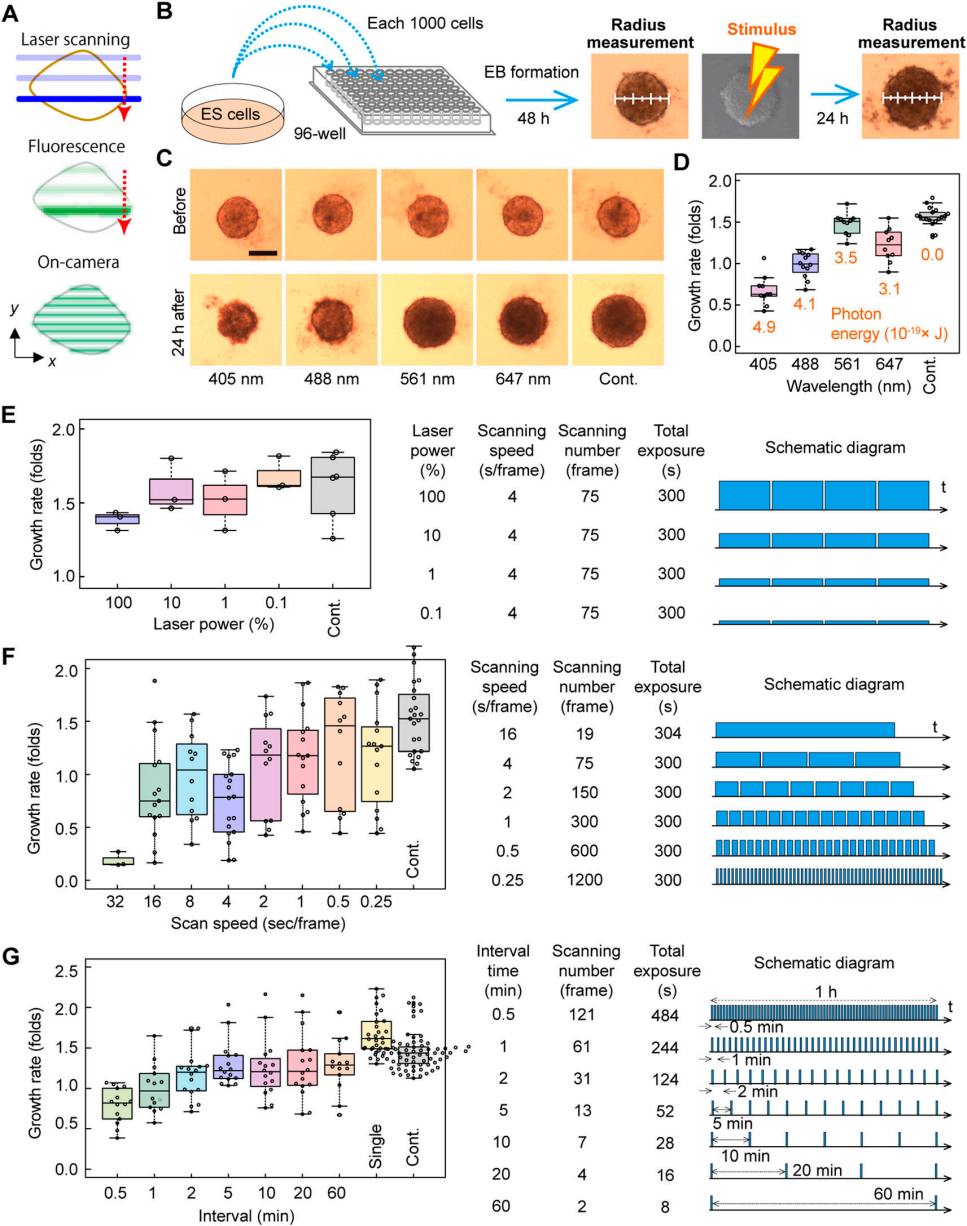
Investigation of phototoxicity using embryoid body (EB) growth and a confocal microscope. **(A)** Schematic illustration depicting the formation of light sheets in the current method. **(B)** Explanation of the assay protocol for investigating EB phototoxicity. The growth rate of an EB was estimated from the ratio of the EB’s area before and after photostimulus using confocal scanning. **(C)** Example of phototoxicity assay and excitation dependence. Bright-field images of each EB were recorded before (*upper*) and 24 h after (*lower*) photostimulus via confocal scanning (75 times with a scan speed of 4 s/frame without intervals). The scale bar is 200 μm. **(D)** Box plot presenting the EB growth rate defined by the ratio of EB diameters before and 24 h after stimulation with various wavelengths. The values in the graph indicate the photon energy received by an EB because of photostimulus. Each circle indicates a single EB. **(E, F, G)** Box plots depicting the scan speed dependence on excitation power (E), growth rate (F), and interval time between two frames (G). On the right are the estimated growth rates, and on the left are the conditions and schematic diagrams of each assay. For (F), the scan speed was fixed at 4 s/frame. Each circle indicates a single EB. The control (*Cont.*) represents data stimulated by the same scanning procedure with 0% output of the laser (*gray*).

Phototoxicity resulting from continuous laser excitation poses a constraint on the duration of observations involving embryos (12). In this study, we delved into the impact of laser irradiation on ESCs. Although the use of a cylindrical lens represents a cost-effective and straightforward method to shape the laser into a thin sheet, it induces a barrel-shaped sheet, leading to an optical cross-section with nonuniform spatial thickness. To address this, we employed a method where a focused laser beam is scanned in parallel to create a pseudo-light sheet with a uniform thickness along the scanning direction (18) (Fig 2A). Consequently, the parameters that had to be optimized during observation include total laser power, scan speed, and interval time between frames, mirroring considerations in confocal observation.

Because laser sheet formation was achieved using the same method as for confocal scanning, we used a confocal microscope to simplify evaluation of phototoxicity for early development. Although it is preferable to use the developed SPIM to investigate cellular phototoxicity during observation with the same system, the specialized sample holder of our developed SPIM rendered it impractical for routine experiments. Phototoxicity in ESCs is typically assessed using propidium iodide (PI) staining (19). However, similar to embryo, limited dye penetration and reduced light transmittance in EBs further hindered viability assessment, often requiring enzymatic dissociation and flow cytometry for reliable measurements (20). In the above context, we developed a reproducible, quantitative, and straightforward evaluation assay for low-dose phototoxicity, using the growth rate of an EB before and after photostimulus via focused laser scanning as an index (Fig 2B; see the Materials and Methods section for details). After photostimulus for the acquisition of 75 images, the growth rate exhibited a correlation with the stimulation photon energy, with the exception of the red laser. Interestingly, the red laser induced more damage than the green laser (Fig 2C and D). This result suggests that longer wavelengths do not necessarily translate to lower toxicity.

Continuing our investigation, we systematically screened observation parameters—laser power, scan speed, and interval time between two frames—related to phototoxicity. The laser wavelength remained fixed at 488 nm, consistent with embryo observation. Remarkably, the growth rate of EBs exhibited a clear negative correlation with laser power (Fig 2E).

**Figure S1.**
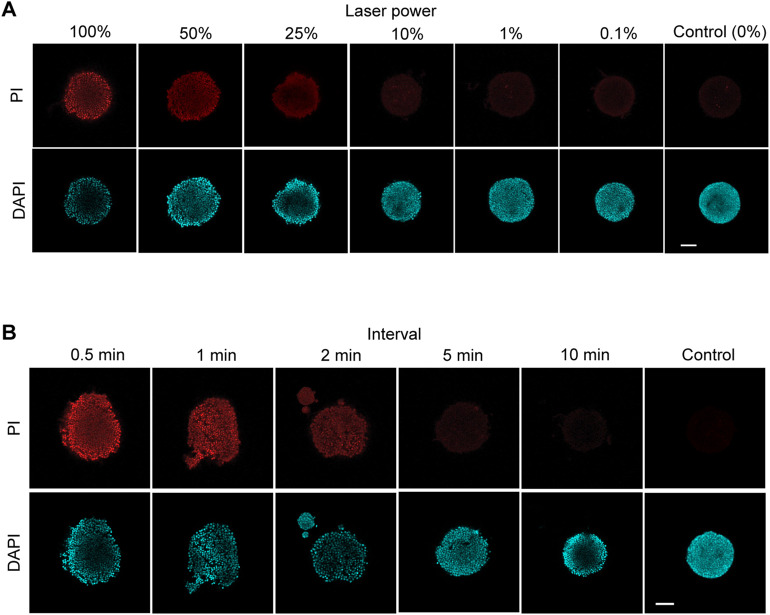
Cell death assay of phototoxicity against EBs using propidium iodide (PI) staining. Typical results of cell death assay at varying laser scanning conditions using EBs. **(A, B)** Laser power (A) and interval time (B) dependence during sequential scanning on phototoxicity to EBs. EBs formed from E14tg2a mouse ESCs were cultured in DMEM supplemented with LIF and 10% FBS in a glass-bottomed dish for 24 h. The EBs were exposed to repeated illumination using a confocal laser scanning microscope (Nikon TiE-A1RSi) equipped with a 20× objective lens (Nikon CFI Plan Apo VC 20×) and a 488-nm laser for 300 s. The illuminated area measured 645 × 645 μm, corresponding to 512 × 512 pixels. After photostimulation, dead cells were selectively stained with PI (19174-31; Nacalai). After 180 min of incubation for PI staining, EBs were fixed with 4% PFA in PBS and counterstained with 4′,6-diamidino-2-phenylindole (DAPI; DOJINDO, 342-07431) in PBS containing 0.1% Triton X-100. The scale bars are 100 μm.

When maintaining a constant total exposure time and laser power, we observed a negative correlation between the growth rate and scan speed (or a positive correlation with frame number), even with a consistent total photon energy delivered to the cells (Fig 2F). In essence, when aiming for the same number of frames, higher scan speeds were associated with lower phototoxicity. Importantly, with a fixed scanning speed, the growth rate showed no significant difference between the 5- and 60-min intervals in this assay. Although qualitative, comparable result has been observed in cell death assays using PI staining (Fig S1). This suggests that the reduction of phototoxicity by decreasing the frame rate reached saturation at the 5-min interval condition, despite the total number of photons changing by a factor of nine (Fig 2G). This result emphasizes that a smaller frame number does not necessarily lead to reduced phototoxicity and underscores the existence of an optimal condition for minimizing damage and maximizing the frame rate.

### Investigation of correlation between phototoxicity and ROS production

**Figure 3.**
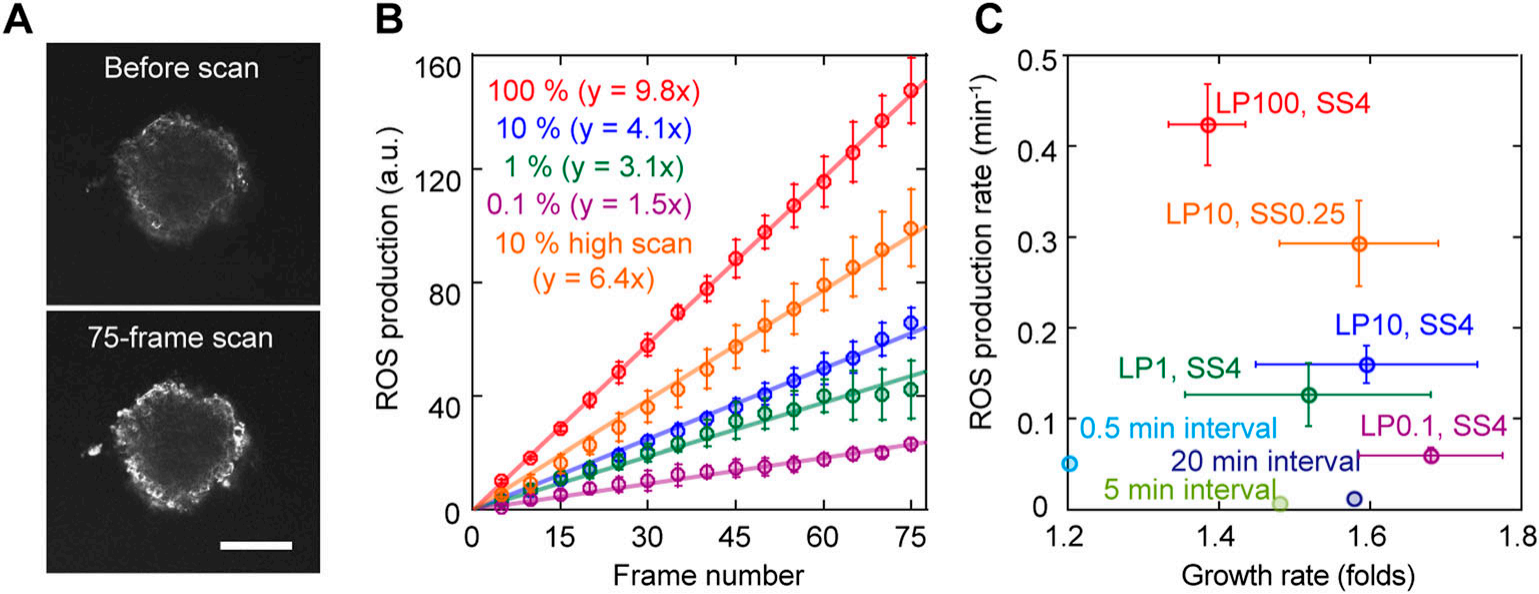
Evaluation of ROS production in an EB during confocal fluorescence imaging. **(A)** Typical confocal fluorescence images of a reactive oxygen species (ROS)–sensitive dye in an embryoid body before (*upper*) and after (*lower*) excitation by 100% output of a 488-nm laser. The image contrast was normalized to that before scanning. The scale bar is 100 μm. **(B)** Time course of estimated ROS production during confocal fluorescence imaging with a scan speed of 4 s/frame excited by 100% (*red*), 10% (*blue*), 1% (*green*), and 0.1% (*magenta*). The slope of the linear approximation indicates the ROS production rate (*lines*). **(C)** Correlation between the ROS production rate and the EB growth rate during confocal fluorescence imaging with a scan speed of 4 s/frame excited by 100% (*red*), 10% (*blue*), 1% (*green*), and 0.1% (*magenta*). The error bars represent the SD. LP, excitation laser power; SS, scan speed. **(B, C)** Data when setting the scan speed to 0.25 s/frame and excitation laser power to 10% overlap in (B, C) (*orange*).

One of the primary contributors to phototoxicity induced by laser irradiation is attributed to ROS production (12). In this study, we delved into ROS production in cells induced by laser scanning. The relative ROS production was estimated based on the fluorescence intensity of an ROS-sensitive fluorescent probe introduced into an EB (Fig 3A). ROS accumulation in cells increased with each stimulus, depending on the laser power (Fig 3B). Notably, the correlation between laser power and the estimated production rate was distinctly nonlinear (0.42 ± 0.04 for 100%; 0.16 ± 0.02 for 10%; 0.13 ± 0.03 for 1%; 0.06 ± 0.007 min⁻^1^ for 0.1%).

Increasing the scan speed from 4 s/frame to 0.25 s/frame resulted in only about a 1.5 times promotion of ROS production (0.29 ± 0.05 min⁻^1^), despite a 16-fold increase in the total number of frames (Fig 3B, orange). This aligns with our previous finding that the faster the scan speed, the lower the phototoxicity (Fig 2F). The estimated ROS production rate showed a negative correlation with the EB growth rate concerning laser power dependence (Fig 3C, red, blue, green, and magenta), highlighting a clear relationship between ROS production and phototoxicity. In contrast, the interval significantly inhibited ROS production, whereas the growth rate did not recover during the interval (Fig 3C, cyan, light green, and dark blue), suggesting the presence of another factor contributing to phototoxicity besides ROS production.

**Figure S2.**
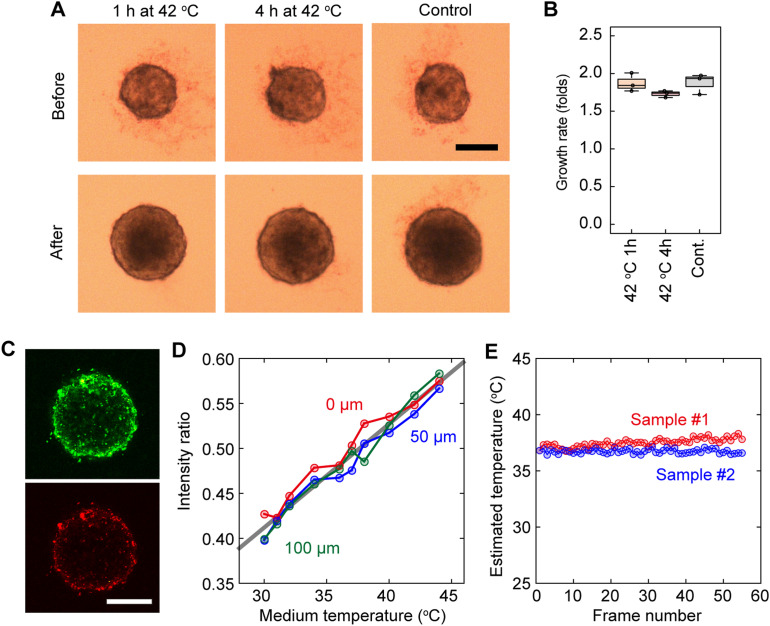
Temperature dependence of the EB growth rate. Bright-field images of an EB before (*upper*) and after (*lower*) exposure to a temperature stimulus instead of a photostimulus. After EB formation, the EB was cultured for 24 h in conditions of 37°C, 5% CO_2_, and >95% RH (*right*). The temperature was increased to 42°C for either the first 1 h (*left*) or the first 4 h (*middle*) of the 24-h period. The scale bar is 200 μm. **(B)** Box plots showing the estimated EB growth rate after a 1-h temperature increase (*left*), a 4-h temperature increase (*middle*), and no temperature increase (*right*). **(C)** Fluorescence images of an EB labeled with a dual-color thermoprobe, DBThD-AA (*upper*) and BODIPY-AA (*lower*). The scale bar is 200 μm. **(D)** Dependence of the intensity ratio of DBThD-AA fluorescence and BODIPY-AA fluorescence on solution temperature at the surface of an EB (*red*), a depth of 50 μm (*blue*), and a depth of 100 μm (*green*) in the EB. The temperature inside the EB could be estimated using the calibration curve obtained from the plots (*gray line*). The ratio remained consistent with the medium temperature, independent of the observation depth. **(E)** Typical time course of the temperature at a depth of 100 μm in an EB during sequential image acquisition with a scan speed of 4 s/frame and excitation laser power of 100%. The temperature increase was less than 1°C, even at its maximum.

Although intense laser irradiation poses a risk of increasing the temperature inside an EB, potentially causing cellular toxicity as reported previously (21), our experimental conditions did not result in a temperature increase within the EB to the extent that hindered growth under continuous laser excitation (Fig S2).

The present results indicated that the correlation observed between ROS production and the growth rate under specific conditions is just one facet of the intricate mechanism of phototoxicity during microscopic observation. The mechanisms underlying acute phototoxic injury, such as cell death, are relatively straightforward to elucidate through scientific experiments because photostimulation directly disrupts critical proteins essential for cell function. In contrast, acquired phototoxicity from low-dose exposure involves a complex interplay of factors, including direct damage to DNA or proteins, the dynamics of ROS metabolism and recovery pathways, and self-induced oxidative stress (22). Therefore, identifying the exact underlying mechanism remains challenging. We speculated that the capacity of a cell to repair damage plays a pivotal role in mitigating phototoxicity. Reducing laser scan speed decreases localized irradiation time, potentially allowing repair pathways to counteract the damage. Although the hydroxyl radical reaction, the most potent ROS, has a remarkably short half-life (∼10⁻⁹ s) (23), ROS production depends on the balance between ROS generation and decomposition. At a faster scan speed of 0.25, ROS levels only increased 1.5-fold compared with a scan speed of 4, despite a 16-fold higher total photoexposure (Fig 3C). Until a comprehensive understanding of phototoxicity mechanisms is achieved, optimization remains reliant on actual measurements. As the growth rate serves as a measurable phenotype of developmental processes, it offers a practical and quantifiable indicator of phototoxic effects during development.

### Long-term SPIM observation of an E5.5–E6.5 embryo

**Figure S3.**
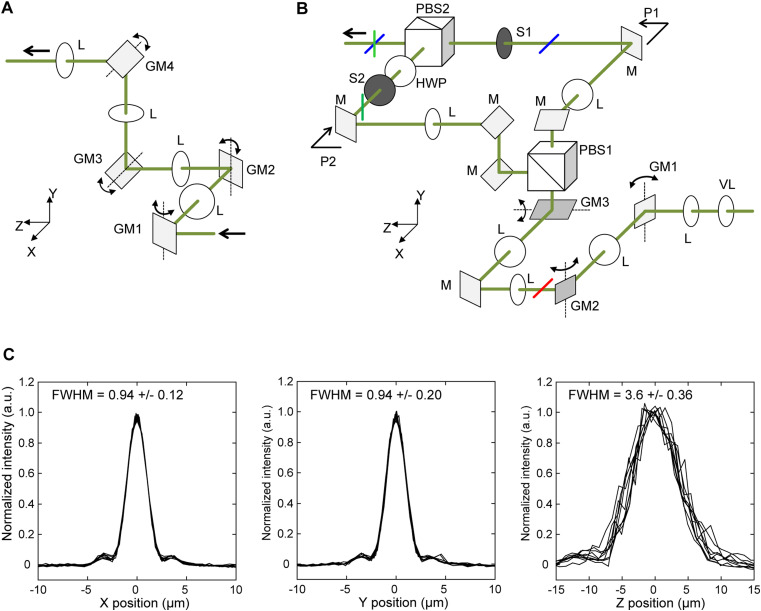
Optical setup for parallel use of mSPIM and diSPIM. **(A)** Implementation of a current optical system with successive optical elements to generate two orthogonal light sheets. GM1 and GM3 are positioned at conjugate focal planes, whereas GM2 and GM4 are positioned at conjugate pupil planes. **(B)** Optical system with a bifurcated 4f configuration was developed and used in the present study, symmetrically placed on both sides of the incubation box. GM1 is positioned at a conjugate focal plane, whereas VL, GM2, and GM3 are located at pupil planes. The red, blue, and green solid lines represent the scanning directions of the laser beam at each point. **(C)** Intensity profiles of a fluorescent microsphere along the x- (*left*), y- (*center*), and z-axes (*right*). Ten measurements were obtained along each axis. FWHM refers to the full width at half-maximum of the fluorescence peak around the microsphere. GM, galvanometer mirror; HWP, half-wave plate; L, plano-convex lens; M, dielectric multilayer mirror; PBS, polarized beam splitter; S, mechanical shutter; VL, varifocal lens.

According to the findings from phototoxicity investigations, maximizing the scan speed effectively reduced phototoxicity. In this study, we optimized the exposure time for a single image acquisition, setting it as short as possible (100 msec), in which the three-directed light sheet was formed with 0° and ±6° scanning at maximum speed (33.3 msec for each directed sheet). In addition, we implemented an optical setup to expedite the 90° rotation of the light sheets and adjust the focus position (see the Materials and Methods section, and Fig S3). The total number of frames for a 3D image reached 600 frames (300 frames along each axis).

**Figure 4.**
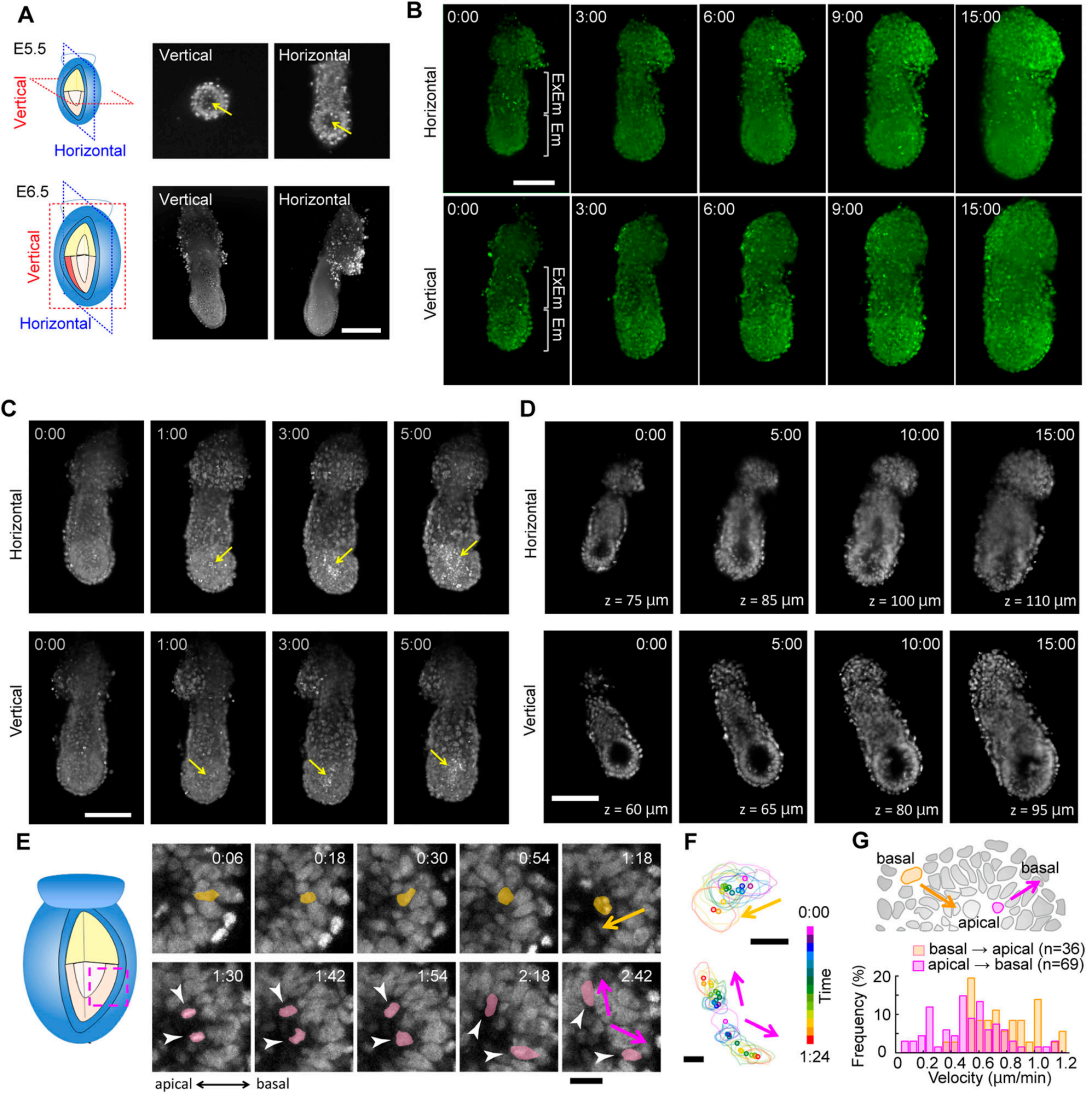
Long-term observation of a mouse E5.5 embryo. **(A)** Typical example of dual-view imaging of an *R26-H2B-EGFP* mouse embryo at E5.5 (*upper*) and E6.5 (*lower*). Left, schematic; middle, vertical image; right, horizontal image. The scale bar is 100 μm. Arrows indicate the cavity. **(B)** Maximum intensity projection snapshots of the time-lapse images of an *R26-H2B-EGFP* mouse at the E5.5 stage. 49 horizontal and 45 vertical images with a 5-μm z-step were acquired every 6 min for 15 h. The scale bar is 100 μm. Em, embryonic region; ExEm, extraembryonic region. **(C)** Maximum intensity projection of the time-lapse images of an *R26-H2B-EGFP* mouse embryo at E5.5. 61 horizontal and 71 vertical images with a 5-μm z-step were acquired with high-power laser excitation every 5 min for 5 h. Yellow arrows indicate cell debris. The scale bar is 100 μm. **(D)** Optical sections of the time-lapse images of an *R26-H2B-EGFP* mouse embryo at E5.5 in the deepest region. The scale bar is 100 μm. The z-value represents the observation depth. **(E)** Snapshots of the time-lapse images of optical sections. A cell nucleus in the epiblast moves to divide near the proamniotic cavity (*yellow*), and each daughter cell intercalates into different positions in the epiblast (*magenta*). The scale bar is 20 μm. **(F)** Tracking result of cells in (E). Lines represent cell outlines; dots represent the weight center. Upper, mother cell; lower, daughter cells. The scale bar is 10 μm. **(G)** Distribution of the velocity (μm/min) of INM-like movement from basal to apical (*yellow*, mean ± SD, 0.81 ± 0.24, n = 36) or from apical to basal (*magenta*, 0.56 ± 0.25, n = 69).

Continuous image acquisition at intervals of 5 min for a duration of 24 h maintained the normal development of a mouse embryo from E5.5 to E6.5 (Fig 4A and B and Video 1, Video 2, Video 3, and Video 4). However, elevating the irradiation power 10-fold resulted in the cessation of embryo growth, leading to a reduction in size (Fig 4C and Video 5 and Video 6). In normal development at E5.5, a cell-less region known as the cavity was observed (Fig 4A, yellow arrow). Contrastingly, during continuous time-lapse imaging with 10-fold increased irradiation power, cell debris appeared in the epiblast within 1 h and gradually accumulated in the amniotic cavity (Fig 4C, arrows). Thus, the phenotype of a mouse embryo experiencing phototoxicity was easily discernible through long-term observation using the present microscope system.

Video 1 Maximum intensity projection of the time-lapse images of the *R26-H2B-EGFP* mouse embryo at E6.5, related to Fig 4A. Images were acquired every 5 min for 18 h. Download video

Video 2 Optical sectional time-lapse images of the *R26-H2B-EGFP* mouse embryo at E6.5, related to Video 1. Images were acquired every 5 min for 18 h. Download video

Video 3 Maximum intensity projection of the time-lapse images of an *R26-H2B-EGFP* mouse embryo at E5.5, related to Fig 4B. Images were acquired every 6 min for 15 h. Download video

Video 4 Optical sectional time-lapse images of the *R26-H2B-EGFP* mouse embryo at E5.5, related to Video 3. Images were acquired every 6 min for 15 h. Download video

Video 5 Maximum intensity projection of the time-lapse images of the *R26-H2B-EGFP* mouse embryo at E5.5, damaged by high-power laser irradiation. Images were acquired every 5 min for 20 h. Download video

Video 6 Optical sectional time-lapse images of the *R26-H2B-EGFP* mouse embryo at E5.5, damaged by high-power laser irradiation. Images were acquired every 5 min for 20 h. Download video

Up to 5–6 h after E5.5, all cells could be reliably isolated using a snapshot of a 3D stack of the image at either plane or a 3D rendered image constructed from dual-axis image stacks (Fig 4D, *left and second left*). However, after 10–12 h, it was difficult to isolate single cells with only a snapshot (Fig 4D, *second right*). Taking temporal information into account, single-cell isolation was still possible in the ectoplacental cone and the extraembryonic visceral endoderm, whereas some cells in the extraembryonic ectoderm could no longer be isolated (Fig 4D, *right*). In summary, we conclude that the present system enables in-toto single-cell isolation in a hemispherical E5.5 embryo, limited to ∼12 h.

Interkinetic nuclear movement (INM)–like migration is thought to be driven by a fundamental mechanism that transcends the developmental stages. With the acquired images, we confirmed the existence of INM-like migration previously reported (8) (Fig 4E). After the division of an epiblast cell at the apical side close to the cavity (Fig 4E and F, yellow marks), the daughter cells intercalated into different parts of the epiblast layer, and their nuclei moved toward the basal side (Fig 4E and F, magenta marks). The mean velocity of INM-like migration in the epiblast was 0.81 ± 0.24 μm/min (n = 36) from basal to apical and 0.56 ± 0.25 μm/min (n = 69) from apical to basal (Fig 4G). These values align with measurements previously taken at the E6.5 stage (8), demonstrating consistency despite structural differences in tissue organization between E5.5 and E6.5.

Thus, the two-layered incubation and laser irradiation protocol facilitated continuous observation of a living mouse E5.5 embryo every 5–6 min for 24 h. However, in-toto single-cell isolation was limited to 12 h, as our optical system, relying solely on linear optics, could not mitigate image degradation in deep biological areas, even with diSPIM compensating for spatial resolution elongation along one orthogonal plane using the other plane. Additional adoption of multiphoton excitation and/or Bessel beam optics should be effective to extend the time limitation of in-toto single-cell isolation (24). Notably, achieving single-cell resolution in the entire E5.5 embryo was realized despite the adverse optical conditions of blue laser excitation. The development of the present microscope system considered usability alongside specification improvement, given that many users still employ GFP as a fluorescent tag or a reporter. Shifting to green-excited red fluorescent proteins, such as mCherry and mKate (25, 26), instead of GFP, is expected to reduce phototoxicity and enhance light permeability.

### New phenomenon found by long-term single-cell tracking in an E5.5 embryo

**Figure 5.**
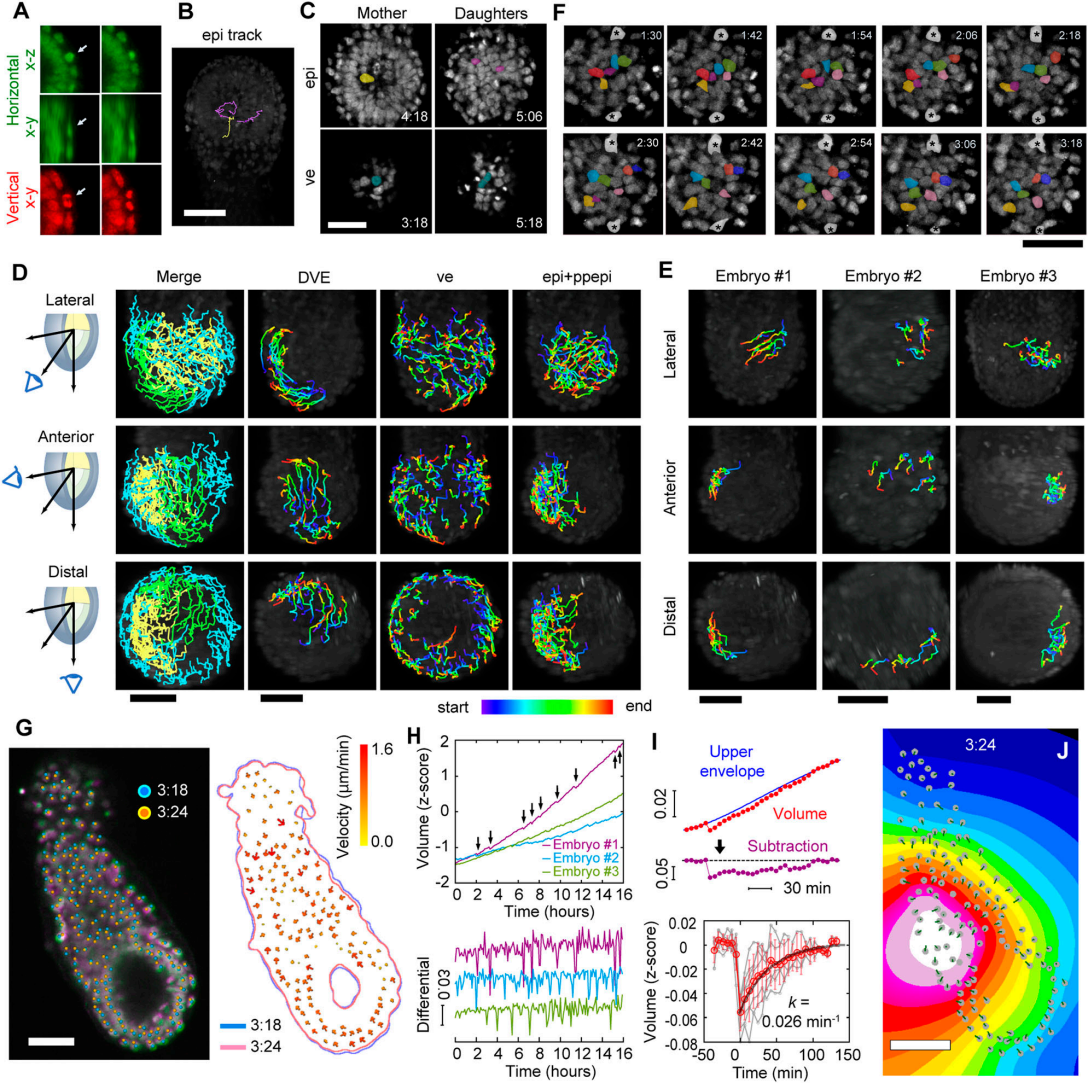
Trans-scale imaging of an E5.5 mouse embryo and analyses of cell movements. **(A)** Snapshots of horizontal (green) and vertical (red) images. The arrow indicates cell division realized in the vertical image, not in the horizontal image. **(B)** Traces of single lineage in the epiblast: a mother cell (*yellow*) and each daughter cell (*magenta*). **(C)** Front view of mitotic cells in the epiblast (*upper*) and the visceral endoderm (*lower*). Left, mother cell; right, daughter cells. **(D)** Tracking results of individual cells during the DVE migration of embryo #1. Left panel (*merge*): green, DVE; blue, visceral endoderm; yellow, epiblast. **(E)** Tracking results of individual cells in the posterior–proximal region excluded from those of the epiblast of each embryo. Spectrum colors in (D, E) indicate elapsed time from the beginning (*blue*) to end (*red*) for 6 h. **(F)** Cell movement in the epiblast of an E5.5 mouse embryo. Colored, epiblast cell nuclei; asterisks, visceral endoderm cells. **(G)** Snapshot of shrinkage of an embryo. The left panel indicates that an embryo at time point 3:24 (*magenta*, elapsed time 204 min) is smaller than that at time point 3:18 (*green*, elapsed time 198 min). Cells at time points 3:18 and 3:24 are represented by cyan circles and orange circles, respectively. The right panel is the tracking results of individual cells. The color and size of an arrow indicate migration velocity from 0.0 to 1.6 μm/min. The cyan and pink lines are outlines of the embryo at time points 3:18 and 3:24, respectively. **(H)** Time course of the volume of an embryo during live imaging. The arrows show the timing of shrinkage. Upper, the z-score, the SD from the mean; lower, the differential of the upper panel. The colors indicate each embryo, that is, #1 (*magenta*), #2 (*cyan*), and #3 (*light green*). **(I)** Hiccup-like behavior after eliminating entire growth. A time course of hiccup-like behavior was obtained by subtraction of the upper envelope from volume data (*upper*). The seven time courses could be extracted (*gray lines*). The traces were set from the moment of shrinkage to time zero and averaged (*red*). Black line, fitting results by a single exponential decay, *f*(*t*) = *a*∙exp (−*k*∙*t*); error bars, SD. **(J)** Visualization of a shrinkage center of embryo #1 at time point 3:24 from the horizontal view. Colors indicate a shrinkage level at a four-bit scale, from white (min) to black (max). The gray circles are cell positions of the embryo, and green lines indicate distances of cell migration within a frame. The scale bars are 50 μm.

**Figure S4.**
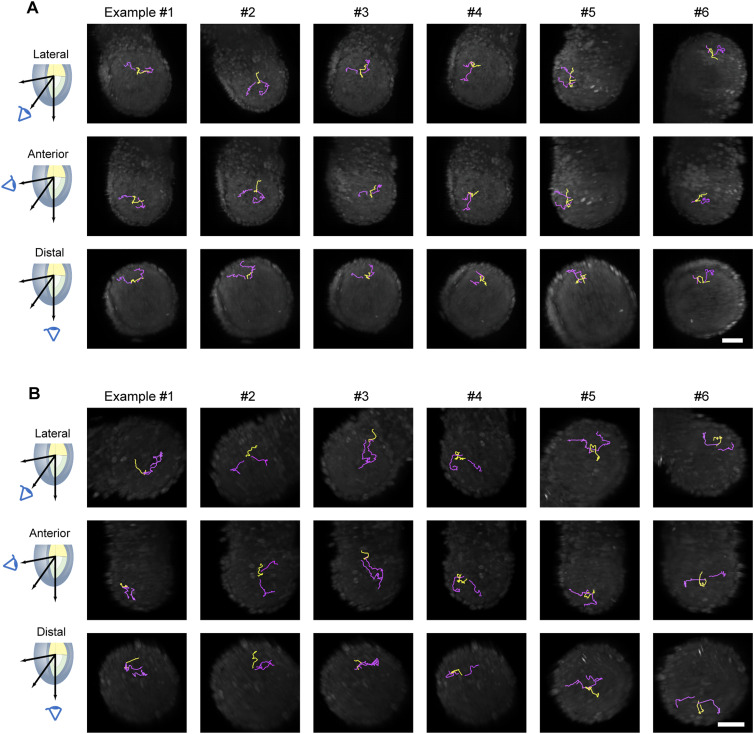
Examples of the migration of a single lineage in the epiblast. **(A, B)** Typical traces of the cell movement, six for each, depicting a mother cell (*yellow*) and its divided daughter cells (*magenta*) in embryos #1 (A) and #3 (B). The scale bars are 50 μm.

In diSPIM, even when achieving single-cell resolution in one axis was not possible, it could be accomplished in the other axis, facilitating a conclusive capture of cell division (Fig 5A and B). This allowed us to perform statistical single-cell analysis. Daughter cells engaged in INM-like migration were notably located apart after their nuclei reached the basal side (71 of 72 lineages, 98.6%), in contrast to daughter cells in the visceral endoderm, which were localized close to each other (155 of 160 lineages, 96.9%) (Figs 5C and S4). Considering the findings of spatial transcriptomic heterogeneity in the epiblast along the distal–proximal axis at E5.25, E5.5, and E5.75 (27), INM-like migration may play a role in rearranging and shuffling cells, contributing to the maintenance of cellular heterogeneity in a tissue or the rapid expansion of the area occupied by fate-determined cells. Further studies, combined with spatiotemporal transcriptomics, will reveal the true nature of this phenomenon.

**Figure S5.**
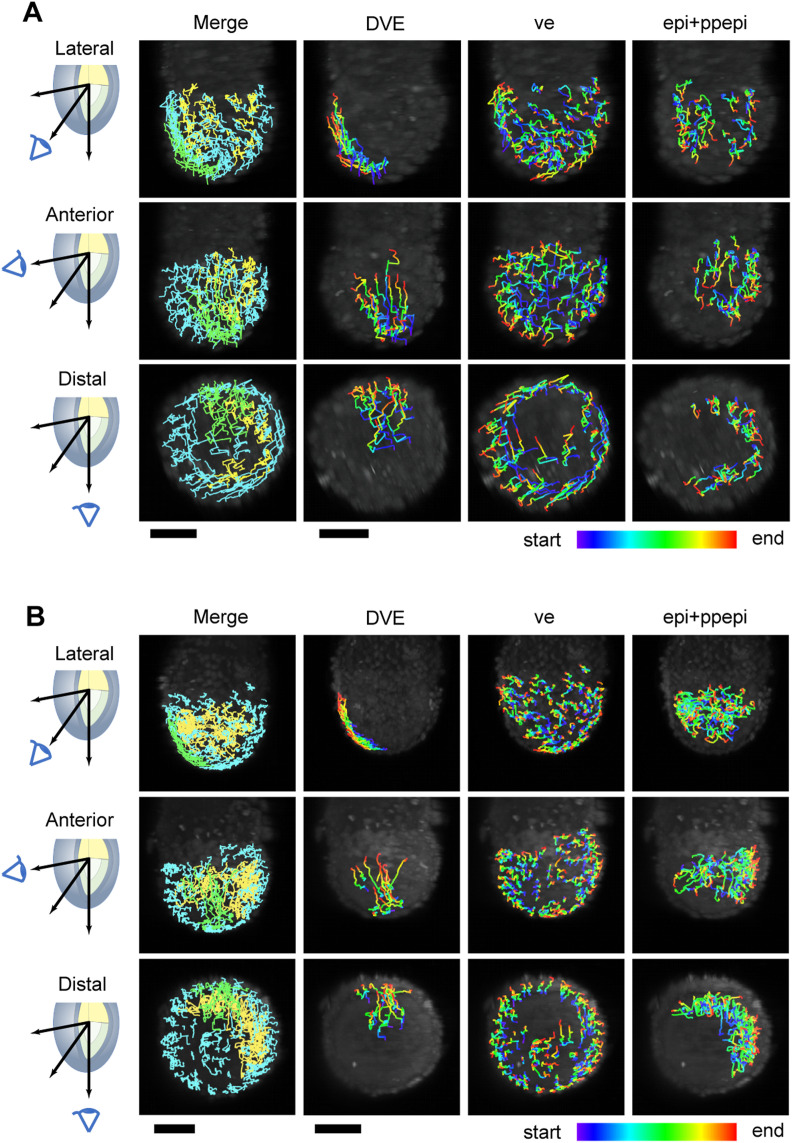
In-toto single-cell tracking in an E5.5 mouse embryo. **(A, B)** Tracking results of single cells during distal visceral endoderm migration of embryos #2 (A) and #3 (B). In the left panel, colors represent distal visceral endoderm (*green*), visceral endoderm (*blue*), and epiblast (yellow). The right three columns show cell tracks in each region using a spectrum color gradient from the start (*blue*) to the finish (*red*). The scale bars are 50 μm.

Because all single cells in a hemispherical embryo were isolated, the cells could be classified into the epiblast, the visceral endoderm, and DVE according to their positions and movement (Fig 5D and Video 7, Video 8, and Video 9). The collective migration of the DVE was successfully identified in all three embryos (Fig S5, *DVE*). During DVE migration, a subset of epiblast cells collectively moved in one embryo (Fig 5E, #1) from the prospective posterior–proximal side to the prospective anterior–distal side close to the visceral endoderm (Fig 5F, colored), whereas visceral endoderm cells exhibited minimal movement (Fig 5F, asterisks). The cessation of DVE migration eliminated the collectivity of epiblast migration in embryo #1, indicating that epiblast cells were passively moved by DVE migration. Meanwhile, we did not observe collective cell movement in the other two embryos (Fig 5E, *#2 and #3*). In addition, the collective movement of visceral endoderm cells in the lateral region, as previously reported (14), was observed in embryos #1 and #2, but not distinctly in embryo #3 (Figs 5D and S5, *ve*). In summary, epiblast and visceral endoderm cells appeared to undergo passive displacement to resolve the distortion in cell arrangement induced by active DVE migration.

Video 7 Cell tracking images of the *R26-H2B-EGFP* mouse embryo at E5.5, related to Fig 5D, from the lateral view. Individual cells were tracked from the elapsed time 0:00–6:00. A 300-min subset of cell tracks is shown by color lines. Green represents DVE, blue is visceral endoderm, and yellow is epiblast. Download video

Video 8 Cell tracking images of the *R26-H2B-EGFP* mouse embryo at E5.5, related to Fig 5D, from the anterior view. Individual cells were tracked from the elapsed time 0:00–6:00. A 300-min subset of cell tracks is shown by color lines. Green represents DVE, blue is visceral endoderm, and yellow is epiblast. Download video

Video 9 Cell tracking images of the *R26-H2B-EGFP* mouse embryo at E5.5, related to Fig 5D, from the distal view. Individual cells were tracked from 0:00–6:00. A 300-min subset of cell tracks is shown by color lines. Green represents DVE, blue is visceral endoderm, and yellow is epiblast. Download video

However, further research is required to validate the observed variations among individual embryos. Current automated single-cell identification methods are incompatible with the dual-axis imaging datasets employed in this study, limiting the number of replicate observations. Although advancements in single-cell analysis technology using artificial intelligence (AI) are inevitable, the application of deep learning requires substantial data. We are now developing a comprehensive automated cell identification system based on AI, which can be retrained with successfully acquired data, to assess the complementary collective cell migrations of the epiblast, DVE, and visceral endoderm, and to understand the mutual/causal relationships among them.

**Figure S6.**
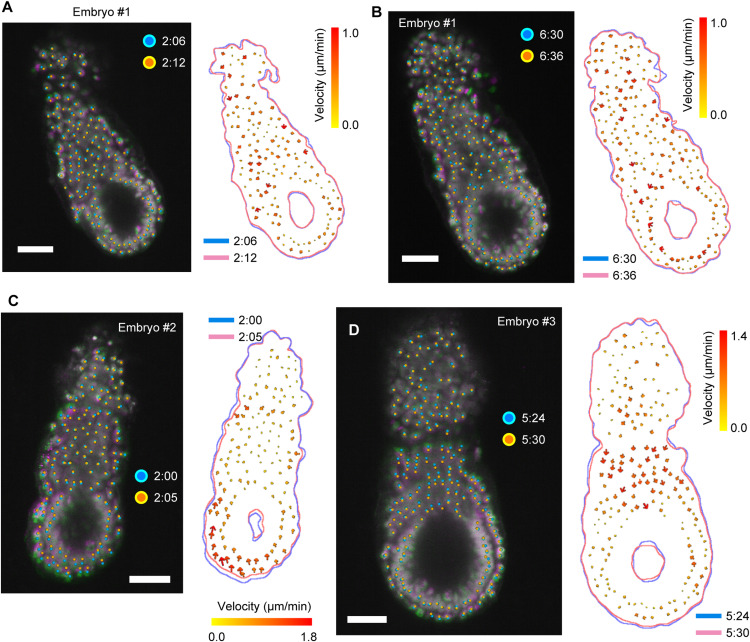
Typical example of hiccup-like behavior in an E5.5 mouse embryo. **(A, B, C, D)** Snapshots capturing the shrinkage process of embryos #1 (A, B), #2 (C), and #3 (D) at two consecutive time points (*first*, *green*; *second*, *magenta*). Cells at the first and second time points are denoted by cyan circles and orange circles, respectively. The right panel illustrates the tracking results for individual cells. Arrow color and size indicate migration velocity, whereas cyan and pink lines represent the embryo outlines at the initial and subsequent time points, respectively. The scale bars are 50 μm.

Moreover, an intriguing behavior in the embryo volume was identified: the embryos exhibited continuous growth but frequently underwent abrupt, hiccup-like contractions (Fig 5G). This hiccup-like behavior was consistently observed in all three analyzed embryos (Figs 5H and S6), with an occurrence frequency of 0.019 ± 0.017 min^−1^ (n = 27). The volume recovered exponentially after shrinkage, and its recovery rate was estimated to be 0.026 min^−1^ through exponential approximation (Fig 5I). Osmotic pressure is critical for the proper internal environment for cellular functions during early development. Water is directed into the lumen of the embryo via an osmotic gradient during the formation of the proamniotic cavity at E4.5 (28). As the cavity grows, the inflow of water is anticipated to increase; however, a rapid change in pressure may cause the blastocyst cavity to collapse (29). According to these previos results, we hypothesize that the abrupt shrinkage regulates the osmotic pressure difference between the interior and exterior of the embryonic cavity, thus stabilizing mechanical signaling in post-implantation embryos.

**Figure S7.**
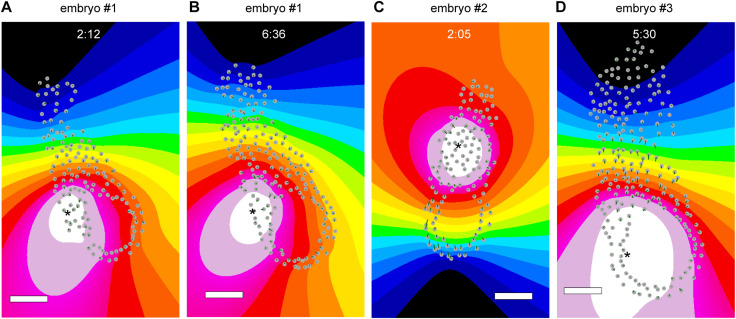
Shrinkage center of hiccup-like behavior in an E5.5 mouse embryo. **(A, B)** Visualization of the shrinkage center of embryo #1 at time points 2:12 (A) and 6:36 (B) from the vertical view. **(C, D)** Visualization of the shrinkage center of embryo #2 at time points 2:05 (C), and of embryo #3 at time points 5:30 (D) from the horizontal view. Colors indicate a shrinkage level on a four-bit scale, ranging from white (min) to black (max). The gray circles represent cell positions of the embryo, and green lines indicate distances of cell migration within a frame. The scale bars are 50 μm.

The spatial and temporal scales of this hiccup-like behavior resembled the abrupt shrinkage of a cavity until the hatching of a pre-implantation embryo, specifically a blastocyst (29). The cavity in an embryo is believed to influence tissue self-organization not only as a mechanical cue but also as a biochemical signaling source, whereas various signal molecules govern cell proliferation and differentiation at E5.5 (4, 30). Therefore, assuming that some mechanical signals might be generated by shrinkage centered on the cavity, we estimated the shrinkage center from the movement of each cell (see the Materials and Methods section for details). Contrary to our expectations, the cavity did not serve as the shrinkage center at E5.5 (Figs 5J and S7), indicating that the hiccup-like behavior we observed was distinct from the abrupt shrinkage observed in a blastocyst.

Despite the same embryo exhibiting the same shrinkage center at different time points (Fig S7A and B), the shrinkage center varied among embryos, yet remained confined to the extraembryonic ectoderm (Fig S7C and D). Osmotic pressure affects embryo size and cell fate (29, 31), and external mechanical stress applied to an embryo promotes A-P axis formation (32). Another hypothesis is that the hiccup-like behavior in an E5.5 embryo may generate chirality in mechanochemical signals, leading to position-dependent cell fate for A-P axis formation. The above hypotheses remain speculative because of the lack of an effective assay system for visualizing osmotic pressure during the live-cell imaging of mouse embryos. Recently, a novel technique using double emulsion droplets was developed, enabling the localized measurement of intracellular and extracellular osmotic pressure within living tissues and successfully facilitating the quantification of spatiotemporal osmotic pressure dynamics during zebrafish embryogenesis (33). We previously developed a genetically encoded intracellular crowding sensor based on a fluorescent protein, which is a candidate indicator of intraembryonic osmotic pressure (34, 35, 36). The fusion of the current method with these advanced technologies will lead to the verification of the hypothesis.

In conclusion, we successfully achieved simultaneous long-term tracking of single cells and the dynamics of tissue formation in a hemispherical embryo for ∼12 h at E5.5. Although the validation of our hypotheses awaits further technological development, the present microscope system visualized each cell migrating toward specific sites, notably one side of the extraembryonic region, for the first time, offering fresh insights into A-P axis formation. The present microscope system is readily accessible for use, and we encourage researchers to employ this system in conjunction with artificial intelligence technologies to comprehensively unravel the multilayered symmetry breaking involved in A-P axis formation.

## Materials and Methods

### Animals

We used *R26-H2B-EGFP* mice (accession no. CDB0238K; https://large.riken.jp/distribution/reporter-mouse.html) (37). The animals were housed in environmentally controlled rooms, and all experimental procedures involving animals were reviewed and approved by the Institutional Animal Care and Use Committee of the RIKEN Kobe Branch.

### Embryo culture

Embryos were harvested and cultured as previously described (38, 39). In summary, embryos at embryonic day 5.5 (E5.5) or E6.5 were cultured in collagen gels (Cellmatrix Type I-A; Nitta Gelatin) mixed with embryo medium. The medium comprised DMEM (D2902; Sigma-Aldrich) supplemented with 50% rat serum, 1 mM β-mercaptoethanol (M-3148; Sigma-Aldrich), 1 mM sodium pyruvate (11360-070; Gibco), and 100 μm nonessential amino acids (11140-050; Gibco). The embryos were cultured in a controlled environment at 37°C and 5% CO_2_. Remarkably, a cultured embryo exposed to suitable irradiation conditions for image acquisition exhibited comparable growth to a similarly mounted embryo on the opposite side of the cuvette.

### Quantification of phototoxicity to an embryoid body

TT2 ESCs (40) were cultured on feeder cells in ES medium (DMEM) (12100-046; Gibco) supplemented with 1,000 U/ml of leukemia inhibitory factor (LIF) (ESG1107; MERCK), 1 mM of β-mercaptoethanol (M-3148; Sigma-Aldrich), 1 mM of sodium pyruvate (11360-070; Gibco), 100 μm of modified Eagle’s medium nonessential amino acids (11140-050; Gibco), and 10% FBS until reaching 70–80% confluence. The ES cells were then collected, suspended in ES medium without LIF (LIF-ES medium), and seeded into a low-adhesion U-shaped-bottom 96-well plate (MS-9096U; SUMITOMO BAKELITE) at a density of 500 cells per well for phototoxicity experiments, 1,000 cells per well for ROS production experiments, and 2,000 cells per well for temperature experiments. EBs formed after two days were used for the phototoxicity experiment, and after a day for the ROS production and temperature experiments. An EB cultured in LIF-ES medium was carefully placed on a glass-bottomed dish (D11040; Matsunami) and positioned on the stage of a Nikon TiE-A1RSi confocal laser scanning microscope equipped with a 20-fold objective lens (CFI Plan Apo VC 20×; Nikon). The EB was then exposed to laser illumination in single scanning mode. The illuminated area measured 645 × 645 μm, corresponding to 512 × 512 pixels. The scan speed was defined as the time per frame (s/frame), which mechanically corresponds to the scanning speed of the galvanometer mirror. After irradiation, the EB was returned to the well and cultured independently for 24 h. Subsequently, images of the EB were captured using Nikon ECLIPSE TS100 equipped with a fourfold objective lens (CFI Plan Apo 4×; Nikon) and a CCD camera (DS-Fi1; Nikon). The cross-sectional area of the image was measured before irradiation (Area.before) and after the irradiation, and again 24 h later (Area.after). The ratio of Area.after to Area.before was calculated to determine the EB growth ratio.

ROS production was estimated by measuring the fluorescence intensity of an ROS-sensitive fluorescent probe (CellROX Deep Red, C10422; Thermo Fisher Scientific) introduced into cells. After a 24-h incubation of EBs with the probe at 37°C, the aforementioned procedure was conducted to assess ROS production.

### Construction of new optics for two-axis light sheets

In the parallel use of mSIPM and diSPIM, we employed two 4f imaging systems, each featuring galvano mirrors (GMs) positioned at conjugate focal planes and conjugate pupil planes, arranged sequentially along a single optical path (refer to Figs 3A and S6A). Nevertheless, achieving identical light sheets on both horizontal and vertical axes proved challenging in practice. Specifically, one light sheet was generated by a GM within the former 4f system (refer to Fig S3A, GM2), whereas the counterpart light sheet was produced by a GM in the latter 4f system (see Fig S3A, GM4). Thus, the practical difficulty arises due to the inherent challenge of precisely matching the characteristics of the two light sheets. The influence of various aberrations introduced by the relay lens along the optical path cannot be overlooked. Notably, the order of the GM responsible for controlling the sheet height (focal plane) and the GM governing sheet formation is reversed between the orthogonal light sheets. Consequently, the orthogonal light sheets are not optically equivalent. Addressing the need to align the waist position of the light sheet with each sample’s position, we developed an innovative optical system, detailed below (see Fig S3).

We incorporated an electrically focus-tunable (varifocal) lens at the conjugate pupil plane (Fig S3B, VL; Optotune) to modify the laser beam’s divergence, thereby adjusting the laser’s focus position. Galvanometric mirrors (Fig S3B, *GM1 and GM2*; Cambridge Technology) were positioned on the conjugate focal plane and the subsequent pupil plane after VL. VL and GM2 were relayed through a 4f system comprising a pair of convex lenses (Fig S3B, *L1* and *L2*; Thorlabs). Another 4f system, consisting of a separate pair of convex lenses (Fig S3B, L3 and L4), was designed with a galvanometric mirror (Fig S3B, GM3; Cambridge Technology) placed on the pupil plane. Subsequently, we constructed a bifurcated 4f system with three convex lenses (Fig S3B, *L5*, *L6*, and *L7*; Thorlabs) and a polarized beam splitter (Fig S3B, *PBS1*; Thorlabs). This bifurcated 4f system generated orthogonal light sheets in the optical paths (Fig S3B, *P1 and P2*). To merge these generated light sheets onto the same pathway, we used a half-wave plate (Fig S3B, HWP) and another polarized beam splitter (PBS2; Thorlabs). Each of the 4f systems, comprising lens pairs (L5, L7) and (L6, L7), relayed the pupil plane at GM3 to the back focal plane of an objective lens. This arrangement facilitated the formation of a light sheet into the sample. To ensure controlled manipulation of the light sheets, optical beam shutters were integrated into each of the branched paths (Figs S1, S2, and S3B). Notably, a scanning motion of GM2 generated one light sheet, whereas scanning GM1 induced the translation of the laser beam incident on GM2, realizing mSPIM. GM3 played a crucial role in controlling the position (height) of the light sheet perpendicular to it. This configuration allowed the two orthogonal light sheets bifurcated by PBS1 to be optically equal and alternately switched by S1 and S2. It is important to note that the irradiating laser used was circularly polarized. The theoretical field of view and lateral resolution of the present SPIM system were calculated based on the Rayleigh criterion, as detailed below. The illumination laser was focused onto the sample using a 10× objective lens (M Plan Apo NIR 10×/NA 0.26; Mitutoyo), and the fluorescence emitted from the sample was collected using a 20× objective lens (Plan Apo L 20×/NA 0.3; OptoSigma). The field of view in the xy-plane measured 660 × 660 μm. By approximating a fluorescence wavelength of 507 nm, the theoretical lateral resolution was calculated to be 1.0 μm. The objective lens used for illumination had a working distance of 30.5 mm and a beam diameter of 3.0 mm entering the lens. Under these conditions, the theoretical diameter of the focused beam at the beam waist was calculated to be 6.6 μm. The axial resolution of a general SPIM system dominantly depends on the light-sheet thickness, rather than the specifications of the detection-side objective, provided that the sheet thickness is sufficiently smaller than the focal depth of the detection objective. If this condition is not met, as in our case, the axial resolution is considered as the interactive product of the focusing performance along the optical axis of the detection objective and the shape of the light sheet (41). The axial resolution is generally smaller than the sheet thickness.

Experimentally, the lateral and axial resolutions were measured as 0.94 μm in the xy-plane and 3.6 μm along the z-axis, respectively (Fig S3C). These values were consistent with those reported in previous studies using a similar optical setup (41). The beam waist of the focused laser forming the light sheet was 7.0 μm, with a sheet width corresponding to a practical field of view of 528 μm. These experimental values were consistent with the theoretical specifications, confirming the precision of the system.

### Configuration of image acquisition and temperature control

A mouse embryo was embedded in a cube (CIDH-29; NK System) containing collagen gels mixed with embryo medium. Subsequently, the cube was then placed at the bottom of a custom glass cuvette with 150–200 μl of embryo medium. The cuvette was then placed in the chamber, and images were captured at 5- or 6-minute intervals using either a 10-fold objective lens (UPlanFL N 10×; Olympus) or a 20-fold objective lens (Plan Apo L 20×; OptoSigma). The imaging setup included a 488-nm laser (JUNO488; Kyocera), a band-pass filter (FF01-525/45-25; Semrock), and a complementary metal oxide semiconductor camera (ORCA-Flash4.0; Hamamatsu Photonics). Laser illumination power was set at about 43 μW on sample.

To ensure optimal equipment performance, room temperature and relative humidity were meticulously maintained at 22.5°C and below 40%, respectively. Room air temperature was regulated at 22.5°C, with additional heating to 37.5°C accomplished using a Tokken TK-0003HU20 heater. The heated air was introduced into the polycarbonate incubation box, encompassing the entire chamber (Fig 1D, magenta). Control of CO_2_ concentration was achieved at 5% through the use of a gas mixture (TK-MIGM01-02; Tokken), specifically within the chamber (Fig 1D, cyan).

To attain elevated humidity levels, 5% CO_2_-enriched air, achieving 95% RT, was introduced into the microscope chamber using a bubbling system (TK-HE05; Tokken) (Fig 1D, orange). In addition, the chamber’s outer frame featured a groove designed to retain water, preventing the evaporation of the medium (Fig 1D, upper right).

### Image registration

To enhance the visual recognition of each nucleus, we combined images acquired in both horizontal and vertical directions. Initially, we equalized image resolutions in the xy- and z-directions. Given resolutions of 0.65 and 5.0 μm/pixel in the xy- and z-directions, respectively, we increased the number of z-slices by 5.0/0.65 times using bicubic interpolation. This equalization process was applied to both horizontal and vertical image stacks at each time point.

Next, image orientations were aligned by three-dimensional rotation of either stack. Subsequently, the image stack underwent registration through spatial shifting or “translation.” Although detailed image registration techniques are elaborated elsewhere (42), we employed Mattes mutual information (43, 44) as the metric and the (1 + 1)-evolution strategy (45) as the optimizer to compute the transformation function. This function was computed for the image stack at the initial time point and applied to all subsequent time points.

Finally, the registered image stack was added in the opposite direction at each time point, creating two fused image stacks. If the horizontal image stack was registered, it was added to the vertical image stack, and vice versa. The all cells within an embryo were manually tracked because current commercially available tracking software was not effective in analyzing these data stacks.

For generating maximum intensity projection images of time-lapse sequences, MetaMorph software (Molecular Devices) was employed. Cell tracking analysis was performed manually using Imaris software (Oxford Instruments).

### Volume computation

To compute embryonic volume, the embryonic region was segmented at each time point. Initially, the image stack underwent denoising using a three-dimensional (3D) Gaussian filter with σ = 0.5 [pixels]. Subsequently, the denoised image stack was binarized through global thresholding, where the threshold was determined by multiplying the mean intensity of the stack by 1.2. Any holes present in the foreground were filled.

Embryonic volume was determined by measuring the number of foreground pixels and converting them to the actual scale. Shrinkage timings were identified as outliers of the volume ratio between two adjacent time points, following the three-sigma rule.

### Visualization of the location of shrinkage

We visualized the coordinates of each image as points where cells approached or receded from abrupt shrinkage. A vector from a specific position *P* to a particular cell *C*_*i*_, where *i* represents the cell number, at a given frame *t* is denoted *PC*_*i*_ (*x*_*i*,*t*_, *y*_*i*,*t*_). The pixel value of a new image at frame *t*, *I*(*x*_*t*_, *y*_*t*_), was determined as the summation of inner products between *PC*_*i*_ (*x*_*i*,*t*_, *y*_*i*,*t*_) and *PC*_*i*_ (*x*_*i*,*t*+1_, *y*_*i*,*t*+1_) normalized by their scalar, as follows:

$$I\left(x_{t},y_{t}\right)=\overset{n}{\underset{i=1}{\sum }}\frac{x_{i,t}x_{i,t+1}+y_{i,t}y_{i,t+1}}{\sqrt{x_{i,t}^{2}+y_{i,t}^{2}}\sqrt{x_{i,t+1}^{2}+y_{i,t+1}^{2}}}$$ (1)

where *n* is the number of observed cells. For visualization purposes, *I*(*x*, *y*) values were plotted on an inverted eight-bit grayscale, and presented using a 16-color look-up table.

## Supplementary Material

Reviewer comments

## Data Availability

All videos and original image data presented in the main results are accessible at ssbd-repos-000243, https://doi.org/10.24631/ssbd.repos.2022.07.243 in SSBD:repository (46). For access to additional videos and analysis results, please contact the corresponding author, who will fulfill reasonable requests.
